# Comprehensive coordinated community based palliative care (C3PaC) model for cancer patients in North India: a mixed-method implementation research study protocol

**DOI:** 10.1186/s12904-023-01184-7

**Published:** 2023-05-23

**Authors:** Mayank Gupta, Ankita Kankaria, Soumya Swaroop Sahoo, Sushma Bhatnagar, Rakesh Kakkar, Anju Grewal, Gegal Pruthi, Lajya Devi Goyal

**Affiliations:** 1 Department of Anaesthesiology, All India Institute of Medical Sciences, Bathinda, Punjab India; 2 Department of Community and Family Medicine, All India Institute of Medical Sciences, Bathinda, India; 3 All India Institute of Medical Sciences, Delhi, India; 4 Department of Obstetrics and Gynaecology, All India Institute of Medical Sciences, Bathinda, India

**Keywords:** Cancer, Comprehensive care, Community, Developing, Low- and lower-middle income country, Implementational research, India, Mixed-method research, Palliative care, Rural

## Abstract

**Background:**

Cancer remains an escalating and challenging public health issue. The management, especially palliative care (PC), is disintegrated and out of reach of in need patients. The overall aim of the project is to develop a feasible and scalable Comprehensive Coordinated Community based PC model for Cancer Patients (C3PaC); congruent with socio-cultural, context and unmet needs in north India.

**Methods:**

A mixed method approach will be used for three-phased pre- and post-intervention study in one of the districts of North India, having a high incidence of cancer. During phase I, validated tools will be used for quantitative assessment of palliative needs among cancer patients and their caregivers. Barriers and challenges for provision of palliative care will be explored using in-depth interviews and focus group discussions among participants and health care workers. The findings of phase I along with inputs from national experts and literature review will provide inputs for the development of the C3PAC model in phase II. During phase III C3PAC model will be deployed over a period of 12 months and its impact assessed. Categorical and continuous variables will be depicted as frequency (percentages) and mean ± SD/median (IQR) respectively. Chi-square test/Fischer test, independent samples Student t-tests and Mann–Whitney U tests will be used for categorical, normally and non-normally distributed continuous variables, respectively. Qualitative data will be analyzed using thematic analysis using Atlas.ti 8 software.

**Discussion:**

The proposed model is designed to address the unmet palliative care needs, to empower community-based healthcare providers in comprehensive home-based PC and to improve the quality of life of cancer patients and caregivers. This model will provide pragmatic scalable solutions in comparable health systems particularly in low- and lower-middle Income countries.

**Trial registration:**

The study has been registered with the Clinical Trial Registry-India (CTRI/2023/04/051357).

## Background

With an estimated cancer prevalence of 2.5 million, and the majority presenting in advanced stages, more than 1 million people need palliative care (PC) in India [1]. Most of these patients present in advanced stages of cancer due to poverty, lack of awareness, poor health-seeking behaviour, and scarcity of cancer diagnostic and therapeutic facilities [2]. Although access to PC is essential to achieve universal health coverage, very few (< 4%) Indians have access to it. This gap exists despite the ongoing National Programme for Prevention and Control of Cancer, Diabetes, CVD and Stroke (NPCDCS) and National Programme for Palliative Care (NPPC) [3]. The lack of healthcare facilities and trained workforce in providing PC make the situation abysmally bad in rural low-to-middle income countries (LMIC) [4, 5]. With 70% of our population residing in rural India with poor access to healthcare services, there is a long-felt need for community-based PC [6]. Community-based PC has been shown to be the least expensive and most cost-effective delivery model [7, 8]. Despite its vast demography and geography, and a huge burden of non-communicable diseases, India has only a handful of community-based PC services. Kerala, the southernmost state of India, alone has about 90% of approximately 1000 PC services in India [9, 10]. Lack of access to quality PC is therefore the norm rather than the exception; ranking India poorly (59th out of 80 countries) in the quality of death index [11]. Most of Kerala’s success can be attributed to the network of community initiatives known as ‘‘Neighbourhood Network in Palliative Care (NNPC)’’ which is the largest community-owned PC service in the world [12]. Various socio-cultural factors (charity, collective efforts, Panapayatt or money exchange, high literacy rate, local self-help groups, community ownership, micro fundraising) responsible for the success of NNPC in Kerala are different in other parts of India (like philanthropic services offered by gurudwaras in the Malwa Region), necessitating PC delivery models congruent with their specific cultural, socioeconomic, disease, demographic, and need differences [13]. The gap in service delivery is well reflected in the joint statement by the Indian Association of Palliative Care and the Academy of Family Physicians of India which in 2018 proposed an urgent need for nationwide community-based PC programs [14]. Lack of healthcare professionals (HCP) training in PC is one of the common reasons for HCP resistance to and poor availability of PC in developing countries [15]. Investment of time and resources in PC training is a recommended facilitator in addressing the shortage of PC workforce ubiquitous in LMICs like India [16]. This exorbitant need- supply mismatch demands development of models targeting both local socio-cultural barriers and PC needs. In order to address this gap, this project has been planned in Bathinda, Punjab, a district in the “Malwa region” with a well-documented high incidence rate of cancer [17, 18].

## Aim

The overall aim of the project is to develop a feasible and scalable Comprehensive CoordinatedCommunity basedPalliative Care model for Cancer Patients (C3PaC) congruent with the socio-cultural diversity and unmet needs unique to the population of interest.

## Methods/design

**Fig. 1:**
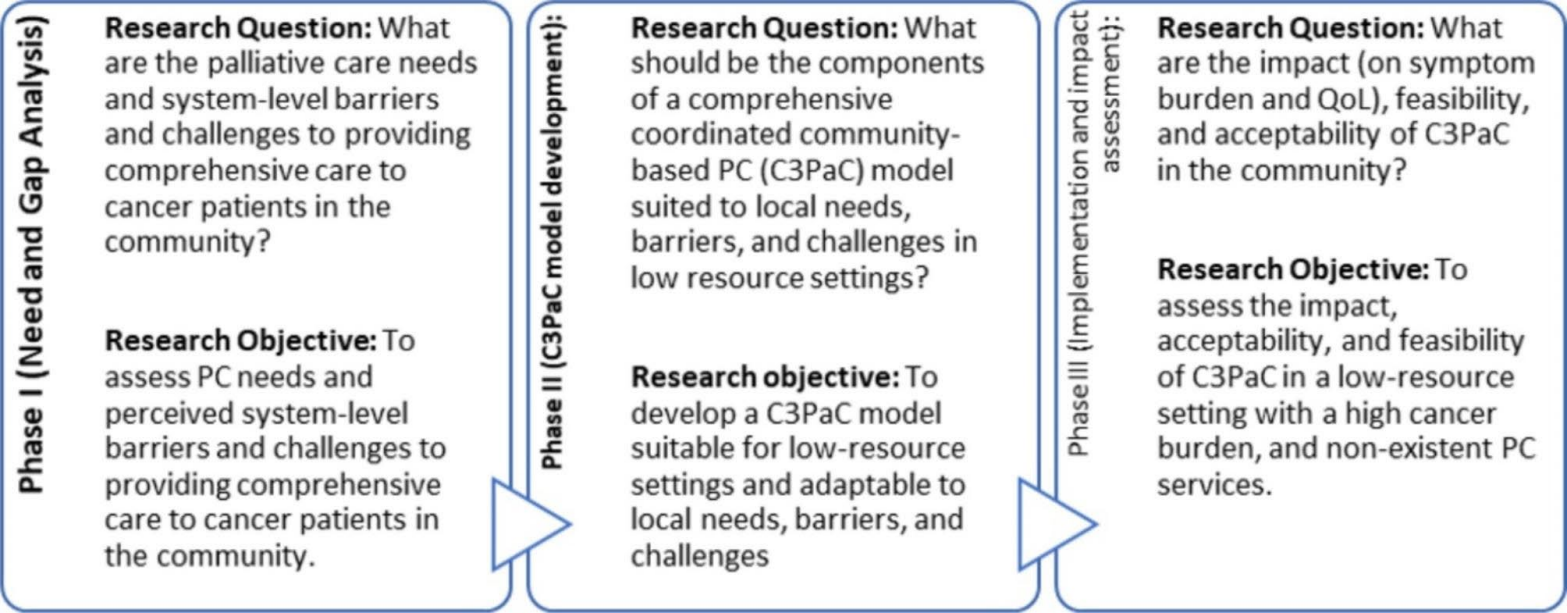
Phase-wise research questions and Objectives

To achieve this aim, a three-phased strategy is planned, each addressing a specific research question and objective (Fig. 1).

### Study design and setting

**Fig. 2:**
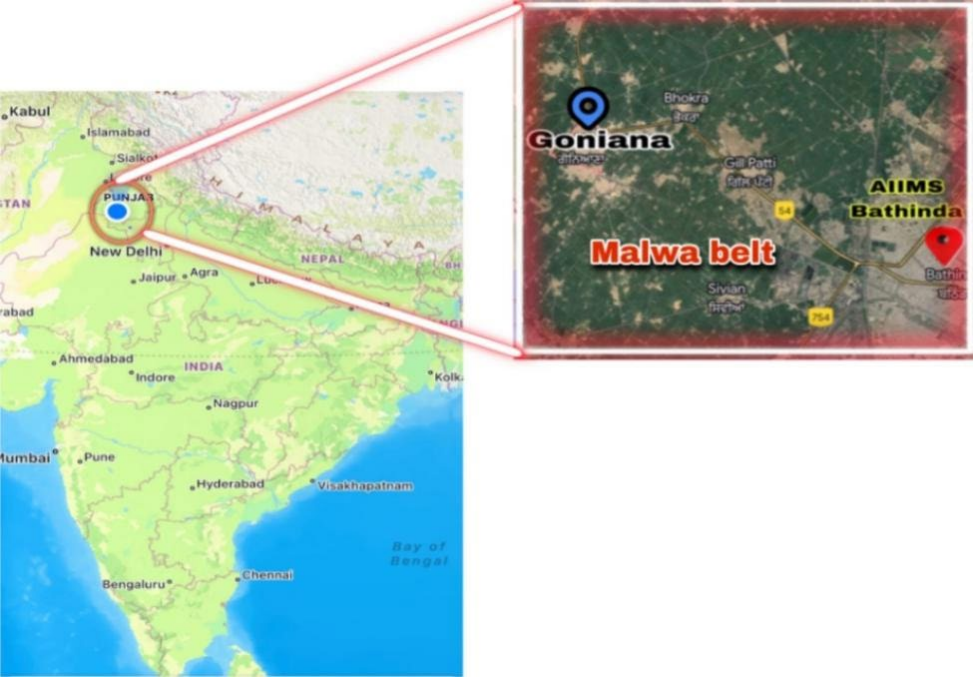
Geographical location of the study site and setting

**Fig. 3:**
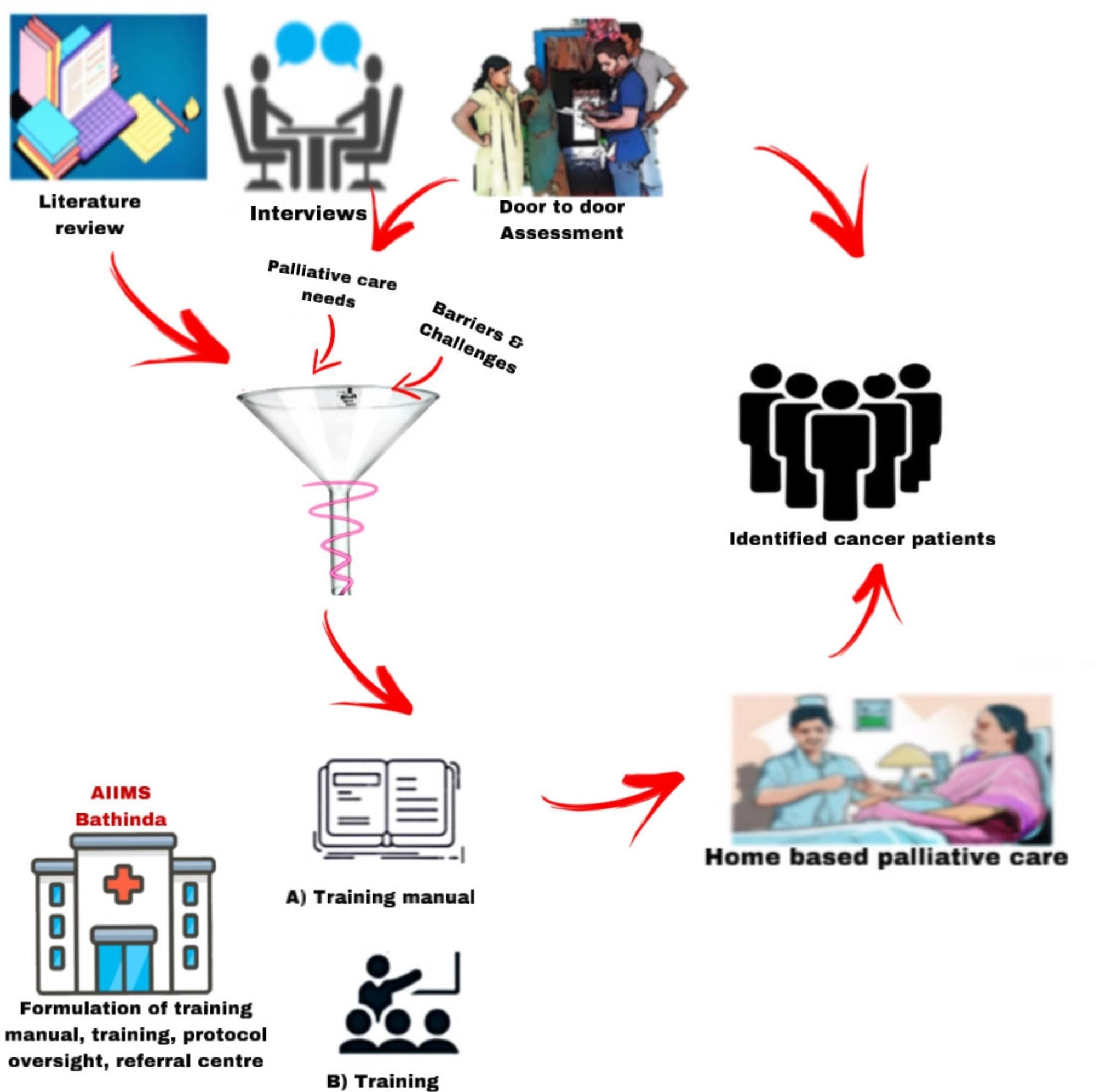
Infographic depicting Project phases

This mixed-methods study will be undertaken by the All India Institute of Medical Sciences (AIIMS Bathinda), a tertiary care institute of national importance in northern India. The study will be implemented in one of the six-blocks (Goniana) of district Bathinda with an estimated population of 2,09,650 (as per the latest district administration report) (Fig. 2). The study area has been selected based on purposive sampling. The study will have following three phases (Fig. 3).

### Phase I (need assessment/gap analysis)

A cross-sectional study with mixed-method design will be adopted. Following activities will be carried out in this phase:

#### Community sensitization workshops

Lack of awareness and knowledge is one of the key factors for suboptimal accessibility to PC [19]. Community sensitization workshops will be held with key stakeholders to orient them about the study and the need and importance of PC [1]. The stakeholders will include panchayati raj institutions (village sarpanch), community health workers like Accredited Social Health Activists (ASHA) and Auxillary Nurse Midwife (ANM), medical officers at the Primary Health Care Centre, medical specialists, and non-communicable diseases (NCD) cell In-charge at the Community Health Care Centre, district program officer of National Programme for Prevention of Cancer, Diabetes, Cardiovascular Diseases and Stroke (NPCDCS), voluntary organizations working in the field of cancer, private providers, and cancer patients. Permission from the concerned authorities will be duly obtained.

#### Key informant interviews

In-depth interviews (IDI)/focus group discussions (FGD) with stakeholders will be conducted to explore the PC needs, barriers and challenges. The interviews will be done using an IDI guide or FGD guide that will be developed in consultation with experts in PC, community medicine, and literature review. About 3 FGDs will be conducted among ASHA workers (tentatively 8 per FGD) and in-depth interviews will be conducted till saturation of data is achieved in other key informant interviews. We will tentatively interview 15–20 patients and their caregivers, 15–20 healthcare providers and 5–7 members of voluntary organizations through purposive and convenience sampling. Adult (18 years or more) patients with diagnosed cancer and their family members involved in patient care will be eligible for the interviews.

#### Door-to-door survey

This will be done by the project staff to identify cancer patients in the community. This exercise will be conducted with the help of ASHA workers. Structured need assessment tools (SPARC-45) and Supportive and Palliative Care Indicators Tool) will be used to allow structured assessment of the PC needs of the identified patients and their families. The supportive and palliative care indicators tool (SPICT) will be used for initial screening followed by the Sheffield profile for assessment and referral to care (SPARC-45). SPARC-45 has been chosen as it allows comprehensive assessment of needs encompassing various domains of PC in various settings [20, 21]. These tools will be adapted, translated into local language and validated for use in local settings after taking the permission.

#### Outcome of phase I

The expected outcome will be to identify the local epidemiology of cancer; symptom burden and experience; unmet needs of cancer patients (and their families) and barriers and challenges to accessing and providing PC. These findings will be helpful in the development of the proposed C3PaC model in phase II.

#### Phase II (development of model)

The C3PaC model will be developed based on the findings of the baseline assessment, expert consensus and literature review and will comprise of [5].

#### Structured training curriculum and training manual

This will be developed in consultation with the national experts (from the Indian Association of Palliative Care; All India Institute of Medical Sciences (AIIMS) New Delhi and AIIMS Bathinda and state and district program officers). Existing guidelines and training manuals in PC will be systematically reviewed and contextualized to the PC needs and training needs of the community health workers. Vernacular translation of the training manual in local languages (Hindi and Punjabi) will be done.

#### Training

The project staff along with the identified community health care personnel from the State Health Authorities, Punjab will be trained at the AIIMS Bathinda after taking permission from the concerned authorities. A multidisciplinary team of experts comprising palliative care specialists, community medicine experts, oncologists, medical social workers, nurses, and psychiatrists will impart comprehensive home-based PC training to potential patients and caregivers. Apart from didactic lectures, field-based experiential training will be provided. Pre- and post-training changes in the knowledge and attitude will be assessed using validated tools.

#### Liasoning within the community and referral pathway

A list of local voluntary organizations and non-governmental organizations (NGOs) working in the area of cancer, palliative care, education and vocational training will be formulated with the help of village heads (sarpanch), community health workers, and self-help groups of the area. Links will be established with these organizations and with cancer treatment centers providing comprehensive cancer-directed treatment to cater to the identified healthcare, physical, financial, and social needs. AIIMS Bathinda will also act as a referral center for patients in need of specialist palliative care services and cancer-directed treatment. Referral coordination, need-based longitudinal follow-up and simultaneous provision of PC will be done by the study team to ensure continuum of care.

### Phase III (deployment of the intervention and feasibility/impact assessment)

A quasi-experimental (pre-post design) will be adopted where the diagnosed patients in Phase I will be considered as controls. To these identified patients, the C3PaC model will be deployed in the form of an intervention over a period of 12 months. The focus will be to provide home-based PC to cancer patients throughout the cancer trajectory, with referral to a tertiary care center for either cancer-directed treatment or management of difficult-to-manage symptoms. The patient information (e.g. demographics, diagnosis, stage of cancer, treatment details, palliative performance scale; physical, psychosocial, and spiritual needs, follow-up details) will be keyed into an electronic record to enable electronic exchange of information among the team members [22].

After one year of intervention, post-intervention data collection will be done to assess the impact, feasibility, sustainability, and scalability of the model using a mixed-method approach. Validated tools like Quality of Life (QOL) version II and the European Organisation for Research and Treatment of cancer Quality of Life Questionnaire Core 30 (EORTC QLQ-C30) will be used after taking appropriate permissions to assess the impact of the C3PAC model on QOL pre and post-C3PAC model deployment. QoL version II has strategically been chosen as their validated translations in the local language are available and both have been used extensively in PC research; thereby improving the generalisability of the results [23–25].

**Fig. 4:**
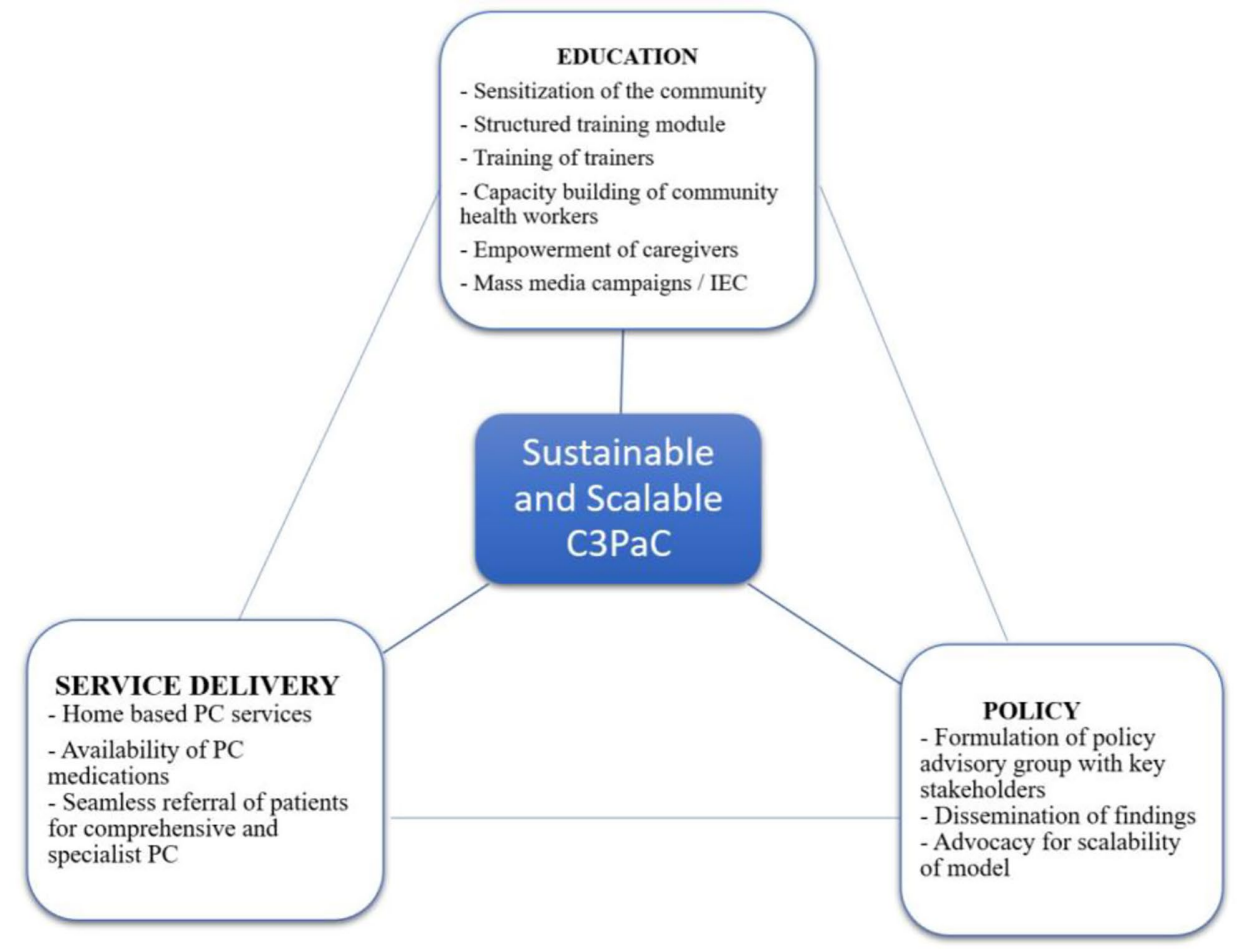
WHO public health strategy for Palliative Care

To explore the feasibility and acceptability of this model, FGD and IDI will be conducted with the key informants (as Phase I). The sustainability of this model will be ensured by using the WHO Public Health Strategy for PC to strengthen health systems (Fig. 4) [1].

### Supervision of the study implementation

A project/policy advisory group (PAG) involving all key stakeholders will be formulated and apprised of the project activities through periodic meetings. The implementation plan will be continuously evolved with the inputs of the PAG. The activities will be periodically supervised and refined over the intervention period.

### Data collection & statistical analysis plan

Data handling will be done with strict confidentiality and proper coding to ensure anonymity. All data including outcome measures will be recorded and stored in an electronic medical record.

#### Quantitative data

A pre-tested semi-structured questionnaire will be used for collecting background information about the study participants and QoL version II will be used to assess the quality of life. The data will be analysed by using the SPSS version 21 (IBM SPSS Statistics for Windows, Version 21.0. Armonk, NY: IBM Corp.). QoL and improvement of symptom score will be taken as the dependent variables while sociodemographic variables (age, gender, education, marital status, mean household income), disease characteristics (primary site of cancer, duration of disease, stage of disease at diagnosis, performance status, comorbidity, symptom assessment, type of treatment received, hospitalisation and health care utilisation) will be taken as independent variables. Categorical and continuous variables will be presented as frequency (percentages) and mean ± SD/median (IQR) respectively. Comparisons between groups will be made using Chi-square test/Fischer test, independent samples Student t-tests and Mann–Whitney U tests for categorical, normally distributed continuous and non-normally distributed continuous variables, respectively. A P-value < 0.05 will be considered statistically significant. Paired t-test or Wilcoxon Test will be applied to measure the difference in mean scores of the study variables (based on the parametric or nonparametric distribution). Linear logistic regression model may be used to determine the predictors of change in mean knowledge scores (pre-training and post-training assessment). If necessary, subgroup analysis will be done.

#### Qualitative data

All the interviews will be conducted by the research team members with expertise in qualitative research, and interviews will be conducted in English, Hindi, or Punjabi (depending upon participants preferences) in a quiet and comfortable setting, ensuring privacy. Field notes will be taken during IDIs and FGDs to ensure conformability. The interviews will be tape-recorded, and transcribed verbatim to text, and interviews conducted in Hindi/Punjabi will be translated into English and then back-translated to ensure appropriateness. Should any participant feels emotionally upset during the interview and decides to opt out, interview will be stopped and counselling session will be offered to him or her. Credibility of the data will be ensured by quality checks, where questions will be rephrased and checks will be done during or at the end of the interviews. An IDI guide and FGD guide will be used and context-specific clarifications will be given to avoid interviewer bias and assure reflexivity. The translated data will be checked for quality and confirmed with language experts of the languages and cultures involved. Data will be coded using deductive and inductive coding. Thematic analysis will be used to analyse the transcripts of implementers and beneficiaries using Atlas.ti 8 software. Both the quantitative and qualitative data will be triangulated.

#### Proposed Outcome of the study

The expected outcomes of the study are tabulated below (Table 1).

**Table 1:** Expected outcomes of the study

Primary outcome	- Development of a feasible and sustainable Comprehensive Coordinated Community based Palliative Care model for Cancer Patients
Services	- % Cancer patients who received PC
Workforce capacity building	- Development of a structured training module for community-based PC. - Number of community health workers trained in PC
Monitoring indicators for evaluating impact of the model	- Change in HRQoL using EORTC QLQ-C30 pre and post C3PAC model deployment. (Permission to use the Hindi and Punjabi versions of EORTC QLQ-C30 has been obtained) - Change in symptoms as measured by an adapted symptom assessment scale. - % Cancer patients cared for at their preferred place of care

## Discussion

Palliative Care interventions (like C3PaC) are inherently complex in that they are multi-component and multi-professional [26, 27]. To ensure their feasibility and cost-effectiveness; these interventions should be developed and tested systematically in phases starting with defining the components, barriers, and challenges to implementation (Phase I); developing the intervention (phase II) and implementation and assessing the effectiveness of intervention (Phase III) [26, 27].

The quality of PC services can be determined by how they address the unmet needs and to be met, they first need to be identified [28]. Need assessment (as in Phase I), inherent in the WHO definition of PC, helps in understanding the requirements of the target beneficiaries enabling them to maintain or improve the present state of well-being [29, 30]. It allows designing appropriate services to provide the right care to those “who” and “when” they need it. A systematic assessment of the quantum of target beneficiaries, their issues, sociocultural milieu, local means available, and existing barriers is essential to delineate the gaps, maximize resource utilization, and empirically plan and implement services [31].

Accustomed culture, lack of training, community awareness, and investment in PC are some of the biggest barriers to the provision of PC; necessitating addressing these barriers (training component of Phase II) to ensure sustainable PC services [20]. Poverty, lack of social security, and most healthcare expenditure being out of pocket in India, necessitate PC programs to cater to free medications, support children’s education, and alternative source of family livelihood (establishing links with voluntary organizations in Phase II) [32].

Based on the principles of universal health care coverage, this study is planned to provide a continuum of care to cancer patients starting from detection, throughout the cancer trajectory, while receiving cancer-directed treatment and extending to end-of-life care. The research team has prior experience in establishing and functioning palliative care programs both at regional and national levels. Since PC provides the desired results when started early, preferably at the time of diagnosis, our model aims at timely initiation of care and integration of the model into the existing healthcare system, with an emphasis on primary care and community setting. Any public health interventions can be ethically challenged if activities cannot be sustained after the cessation of external funding [33]. A public health approach will be adopted to address sustainability throughout the planning, implementation, and evaluation of C3PaC (Fig. 4) [34, 35]. PC activities will be tagged alongside the existing community healthcare system to ensure ownership and continued program activities even after the cessation of the project [32].

### Sustainability and scalability of the C3PAC model

Though the study area is selected based on purposive and convenient sampling based on the proximity to the study hospital, the results of this study would be applicable to the regions with similar health systems and socio-demographic backgrounds. The results will be disseminated and used for advocacy for comprehensive care for cancer patients at the district, state and national level.For sustainability strengthening of primary health care system will be done for PC and referral indicators (when to refer) will be provided to local HCP.

### Strengths

Current literature is grossly deficient in terms of empirical research on effective and sustainable models of PC delivery in rural LMIC [5, 36]. The meager literature has been criticized for lack of precise transparent methodology, data collection, sampling, data analysis and ethical review [5, 19]. To the best of our understanding and knowledge, this will be the first comprehensive evidence-based model of community based PC in rural North India. Our model is unique in that it aims to (1) meet the unaddressed PC needs in the community (2) facilitate early referral of diagnosed cases to cancer care centers for comprehensive management (3) empower healthcare functionaries working in the community in providing comprehensive home-based PC. The collaborative design and coordinated approach will help caregivers and healthcare professionals in implementing palliative regimens in different sociocultural settings and context. It will help policy makers in informed decision making and help them in understanding the challenges in end of life cancer care.

### Anticipated operational difficulties

The scope of PC extends beyond cancer to other chronic life-limiting illnesses. Such a wide definition of the PC population may be impractical, to begin with especially in resource-limited settings [37]. Loss to follow-up due to death or inability to respond to the outcome measures due to cognitive impairment or declining health is common in longitudinal PC studies [25]. This may lead to inadequate statistical power and will be taken into account while calculating sample size [38]. Though challenging, effort will be made to time outcome measures to balance the loss of data due to attrition while allowing sufficient time for the service model to have its effect [39]. The reason for attrition will be systematically analysed, classified (Attrition due to death illness, or Attrition at random), and recorded [24, 40]. Proxy measures will be recorded for patients unable to report themselves [38, 40].

## Data Availability

All the tools used are available for public use and have been cited in text. As this is a protocol paper, no datasets are involved.

## Abbreviations

AIIMS: All India Institute of Medical Sciences
ASHA: Accredited Social Health Activists
ANM: Auxillary Nurse Midwife
C3Pac: Comprehensive Coordinated Community based Palliative Care model for Cancer Patients (C3PaC)
EORTC QLQ-C30: European Organisation for Research and Treatment of cancer Quality of Life Questionnaire Core 30
FGD: focus group discussions
HCP: Health care professionals
IDI: In-depth interviews
LMIC: Low-to-middle income countries
NCD: non-communicable diseases
NGO: Non-governmental organizations
NNPC: Neighbourhood Network in Palliative Care
NPCDCS: National Programme for Prevention of Cancer, Diabetes, Cardiovascular Diseases and Stroke
PC: Palliative Care
QoL: Quality of Life
SPICT: Supportive and Palliative Care Indicators
